# Using Virtual Patients to Explore the Clinical Reasoning Skills of Medical Students: Mixed Methods Study

**DOI:** 10.2196/24723

**Published:** 2021-06-04

**Authors:** Ruth Plackett, Angelos P Kassianos, Jessica Timmis, Jessica Sheringham, Patricia Schartau, Maria Kambouri

**Affiliations:** 1 Department of Applied Health Research University College London London United Kingdom; 2 Primary Care and Population Health Department University College London London United Kingdom; 3 Institute of Education University College London London United Kingdom

**Keywords:** computer simulation, web-based patient simulation, computer-assisted instruction, educational technology, medical education, clinical decision support systems, clinical decision making, clinical reasoning, clinical skills, primary care, diagnosis

## Abstract

**Background:**

Improving clinical reasoning skills—the thought processes used by clinicians to formulate appropriate questions and diagnoses—is essential for reducing missed diagnostic opportunities. The electronic Clinical Reasoning Educational Simulation Tool (eCREST) was developed to improve the clinical reasoning of future physicians. A feasibility trial demonstrated acceptability and potential impacts; however, the processes by which students gathered data were unknown.

**Objective:**

This study aims to identify the data gathering patterns of final year medical students while using eCREST and how eCREST influences the patterns.

**Methods:**

A mixed methods design was used. A trial of eCREST across 3 UK medical schools (N=148) measured the potential effects of eCREST on data gathering. A qualitative think-aloud and semistructured interview study with 16 medical students from one medical school identified 3 data gathering strategies: Thorough, Focused, and Succinct. Some had no strategy. Reanalysis of the trial data identified the prevalence of data gathering patterns and compared patterns between the intervention and control groups. Patterns were identified based on 2 variables that were measured in a patient case 1 month after the intervention: the proportion of Essential information students identified and the proportion of irrelevant information gathered (Relevant). Those who scored in the top 3 quartiles for Essential but in the lowest quartile for Relevant displayed a Thorough pattern. Those who scored in the top 3 quartiles for Relevant but in the lowest quartile for Essential displayed a Succinct pattern. Those who scored in the top 3 quartiles on both variables displayed a Focused pattern. Those whose scores were in the lowest quartile on both variables displayed a Nonspecific pattern.

**Results:**

The trial results indicated that students in the intervention group were more thorough than those in the control groups when gathering data. The qualitative data identified data gathering strategies and the mechanisms by which eCREST influenced data gathering. Students reported that eCREST promoted thoroughness by prompting them to continuously reflect and allowing them to practice managing uncertainty. However, some found eCREST to be less useful, and they randomly gathered information. Reanalysis of the trial data revealed that the intervention group was significantly more likely to display a Thorough data gathering pattern than controls (21/78, 27% vs 6/70, 9%) and less likely to display a Succinct pattern (13/78, 17% vs 20/70, 29%; *χ*^2^_3_=9.9; *P*=.02). Other patterns were similar across groups.

**Conclusions:**

Qualitative data suggested that students applied a range of data gathering strategies while using eCREST and that eCREST encouraged thoroughness by continuously prompting the students to reflect and manage their uncertainty. Trial data suggested that eCREST led students to demonstrate more Thorough data gathering patterns. Virtual patients that encourage thoroughness could help future physicians avoid missed diagnostic opportunities and enhance the delivery of clinical reasoning teaching.

## Introduction

### Background

Clinical reasoning skills are defined as the thought processes used by clinicians to formulate appropriate questions and diagnoses and, therefore, are critical to providing quality health care [1,2]. Poor clinical reasoning skills have been associated with missed diagnostic opportunities and poor patient outcomes [3-6]. To address the need to improve clinical reasoning skills, there has been a call for more explicit teaching of clinical reasoning skills in undergraduate medical education [7,8]. However, the optimal method of teaching clinical reasoning skills is not well understood because of the complexity of the skills and how they vary depending on context, knowledge, and experience [1,2]. Traditional methods of teaching clinical reasoning skills, such as clinical placements, place a considerable burden on faculty time and resources. Furthermore, growing numbers of students can result in fewer opportunities for exposure to a variety of clinical cases [9-11].

The use of digital teaching methods has been recommended to address gaps in clinical reasoning skills teaching and complement traditional face-to-face methods [8,12-15]. Virtual patients, a specific type of computer program that simulates clinical scenarios, has been recommended as an effective method [9,16,17]. Virtual patients allow students to be exposed to a large number of varied patient cases, which can help them develop their knowledge and create more complex mental representations of illnesses [18,19]. Learning through experience, reflection, and deliberate practice can also help students to develop and retain their skills [12,20,21]. Virtual patients are also becoming increasingly similar to clinical practice, as more consultations are being undertaken on the internet [22-25].

### The Electronic Clinical Reasoning Educational Simulation Tool

To address the need for more structured clinical reasoning training using digital methods, electronic Clinical Reasoning Educational Simulation Tool (eCREST) was developed by the authors and web designers, Silver District [26], and is reported in detail elsewhere [27]. eCREST sought to influence 3 cognitive biases that have been found to influence clinical reasoning: the unpacking principle, confirmation bias, and anchoring [28-30]. The unpacking principle is the tendency to not elicit the necessary information to make an informed diagnosis. Confirmation bias is the tendency to seek information only to confirm a diagnosis. Anchoring is the tendency to stick to an initial diagnosis despite contradictory information [31]. To address these biases, eCREST was primarily focused on improving data gathering skills and flexibility in thinking about diagnoses rather than all clinical reasoning skills. In eCREST, students were presented with 3 videos of virtual patient cases. These patients presented to primary care with nonspecific respiratory symptoms, such as cough, which could be indicative of serious conditions such as lung cancer. The students were required to ask the patient questions from a list, received a video response, and formulated diagnoses and a management plan. To address potential biases, reflection was prompted at regular intervals throughout the case by asking students to revise their diagnoses, and they received feedback at the end of each case [32].

A trial evaluated eCREST in 3 UK medical schools to test feasibility and acceptability and is described in detail elsewhere [33]. This trial found that eCREST appeared to influence students’ data gathering but had less impact on flexibility in thinking about diagnoses. Students in the intervention group appeared to show a more thorough data gathering pattern than controls, as they did not miss important information, but there was suggestive evidence (not statistically significant at 5%) that they may ask more irrelevant questions than controls. However, the quantitative data from the trial provided little further insight into how students gathered information while using eCREST and other data gathering patterns. In addition, little is known about how students gather information from previous clinical reasoning studies, as paper vignettes were used that did not require students to gather information [34,35]. A greater understanding of how students gather data when using virtual patients in real time will inform educators about how they can best support students in developing these skills. It could also help developers to design virtual patients that provide better training on data gathering skills. Therefore, this study aims to:

1. Understand how medical students gather information and reach diagnostic judgments when interacting with virtual patients.
2. Identify students’ data gathering patterns while using virtual patients.
3. Examine whether eCREST changes the data gathering patterns of students.

## Methods

### Design

**Figure 1:**
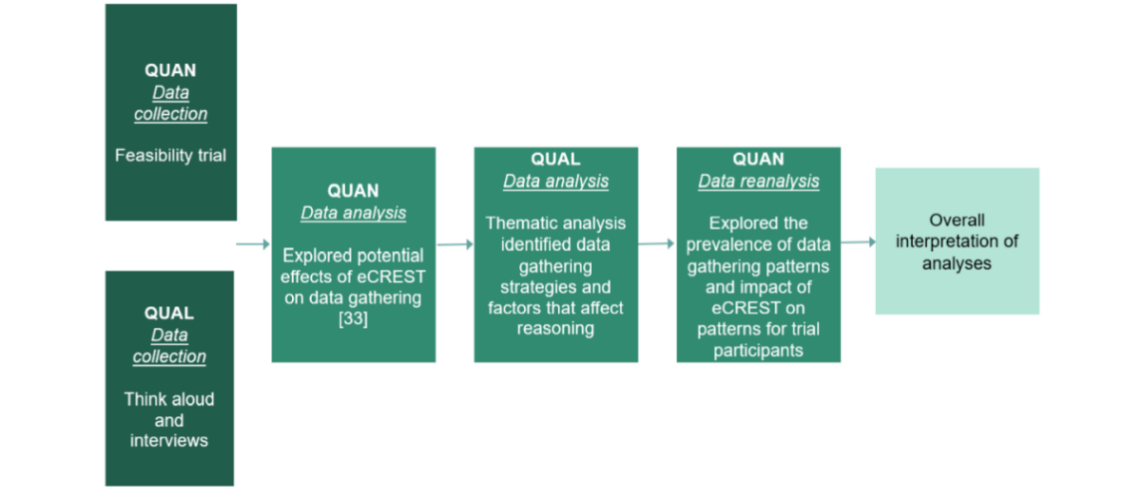
Process of the mixed methods approach. QUAL: qualitative; QUAN: quantitative.

This study used a mixed methods design, as shown in Figure 1 [36-38]. The quantitative method was of equal priority to the qualitative method, and data collection was carried out concurrently and analyzed sequentially. Initially, a trial of eCREST was conducted to explore its potential effects on data gathering. The results of the trial’s feasibility and effects on data gathering are reported elsewhere [33]. The methodological details of this study are also summarized in the sections below. During the trial, think-aloud protocols captured students’ reasoning during eCREST and their reflections on the task. Following the initial analysis of trial data, qualitative data were thematically analyzed and distinct planned strategies of gathering data and factors that affect these strategies were identified (aim 1). This led to a reanalysis of the trial data to identify the prevalence of different data gathering patterns or behaviors among trial participants (aim 2). Finally, an exploratory analysis was undertaken to examine the effects of eCREST on data gathering patterns (aim 3).

### Setting

The trial took place at 3 UK medical schools, with full details reported elsewhere [33]. The concurrent qualitative study took place at one of the UK medical schools participating in the trial, with students who were not part of the trial. Ethical permission for the qualitative study was granted by the University College London (Ref. 9605/001; September 08, 2017).

### Participants

For the trial, 18.16% (264/1454 of all eligible) final year medical students were recruited and randomly assigned to an intervention or control group. A total of 148 students (78 in the intervention group and 70 in the control group) remained in the trial after 1 month [33]. For the qualitative study, 16 final year medical students were recruited through a peer-assisted learning scheme, where the students opted to undertake a project in medical education, via newsletters and snowball sampling. All students were informed that their data would be anonymized and that their participation was voluntary, and they provided written consent. They were incentivized to participate with a voucher.

### Procedure

#### Quantitative Data Collection and Initial Analysis

In the trial, the intervention group received 3 patient cases via eCREST that they had 1 week to complete while receiving teaching as usual. The control group received teaching as usual without access to eCREST until the end of the trial. Clinical reasoning skills outcomes were compared between the groups after 1 month through an additional eCREST patient case. Multimedia Appendix 1 shows a flowchart of the trial. The results indicated that eCREST may encourage more thorough data gathering patterns [33].

#### Qualitative Data Collection and Analysis

We used a think-aloud study design followed by semistructured interviews [39,40]. The think-aloud tasks involved observing students verbalizing their thoughts in real time while completing one patient case in eCREST. This method can provide insights into the clinical reasoning of medical students, as it provides access to their conscious thought processes [13,39,41,42]. A practice think-aloud task was given before the main task to ensure that students were comfortable with the process. Nondirective prompts, such as *keep talking*, were used if students were silent for a significant amount of time. Semistructured interviews were conducted after the think-aloud task to gather students’ retrospective thoughts [39,40]. A topic guide was developed for the interview and piloted with 2 medical students. Each session took approximately 1.5 hours per student.

All 16 think-aloud tasks and semistructured interviews were transcribed and imported into NVivo Version 12 (QSR International) software [43]. We used thematic analysis following the approach of Braun et al [44] and extended upon by Swain [45]. First, we developed deductive codes based on our research aims and initial findings from the trial data. We then familiarized ourselves with the data and developed inductive codes. A codebook was generated by one researcher (RP). To ensure the validity of the coding framework, 3 additional researchers (MK, APK, and JT) used the coding framework to guide their coding of a transcript and generated their own additional codes. We grouped similar codes into themes. Themes were validated in meetings with a wider research team where discrepancies were discussed, and a consensus was reached. Data were analyzed iteratively until the themes reached saturation. The results relating to how students gathered data (aim 1) informed the identification of students’ data gathering patterns in the larger quantitative data set obtained from the trial.

#### Reanalysis of Quantitative Data

Informed by the qualitative findings, we used data from the trial to estimate the prevalence of 3 data gathering patterns (aim 2) and to understand if eCREST may have changed these patterns (aim 3). Student data on an eCREST patient case completed by both interventions and controls 1 month after registration were used to assess clinical reasoning. As described in the trial paper, we constructed variables from these data to capture elements of clinical reasoning skills, such as data gathering ability and flexibility in thinking [33]. In this study, we focused only on the variables related to data gathering skills, as these were found to significantly differ between groups in the trial and the focus of this research was on data gathering skills. We assessed *essential information identified* (Essential) by measuring the proportion of essential questions they asked the patient and patient examinations undertaken, out of all possible essential examinations and questions identified by experts. This measure captured how complete data gathering was irrespective of whether irrelevant questions were also asked. The *relevance of history taking* (Relevant) was measured by assessing the proportion of all relevant and essential questions they asked the patient and patient examinations undertaken, out of the total number of examinations undertaken and questions asked. This measure was an indicator of the specificity of information gathered. Students could select a total of 70 questions and examinations. Experts defined 29 Essential items as a question or an examination that would change the differential diagnosis of the patient case, allow for differentiation as much as possible between alternative diagnoses, and reveal the key symptoms that might be indicative of sinister diagnoses. A further 10 questions were considered by experts as *relevant,* that is, clinically appropriate to ask but would not reveal key information to derive all important possible diagnoses. The remaining 31 questions were defined as *irrelevant*.

To identify the data gathering patterns displayed by students, the quartiles for the Essential and Relevant variables were calculated. Those who scored in the top 3 quartiles on both variables were classified as having a Focused pattern. Those who scored in the top 3 quartiles for Essential but in the lowest quartile for Relevant were classified as having a Thorough pattern. Those who scored in the top 3 quartiles for Relevant but in the lowest quartile for Essential were classified as having a Succinct pattern. Those whose scores were in the lowest quartile on both variables were labeled Nonspecific. Sensitivity analyses showed that other cutoff values below the lowest quartile had insufficient numbers for each pattern to conduct the chi-square analysis and compare scores between the intervention and control groups. We examined whether the use of different data gathering patterns varied between the intervention and control groups using a chi-square test. Analyses were conducted using STATA Version 15 with *P*≤.05, considered statistically significant [46].

## Results

### Sample Characteristics of Qualitative and Quantitative Studies

Table 1 describes the participant characteristics of those in the trial at baseline and the think-aloud study. In the trial, most participants were 23 to 24 years of age (152/264, 57.6%) and were male (142/264, 53.8%). The age and gender of the think-aloud participants were similar: 81% (13/16) were 23 to 24 years old and 56% (9/16) were male.

**Table 1:** Participant characteristics at baseline in the trial and think-aloud study.

Characteristics		Trial				Think-aloud study
		Intervention group (n=137), n (%)	Control group (n=127), n (%)	*P* value^a^	Whole group (n=16), n (%)	
**Age (years)**						
	20-22	4 (2.9)	1 (0.8)	N/A^b^	0 (0)	
	23-24	73 (53.3)	79 (62.2)	N/A	13 (81)	
	25-26	39 (28.5)	29 (22.8)	N/A	2 (13)	
	27-28	11 (8)	10 (7.9)	N/A	1 (6)	
	>29	10 (7.3)	8 (6.3)	.49	0 (0)	
**Gender**						
	Female	64 (46.7)	58 (45.7)	N/A	7 (44)	
	Male	73 (53.3)	69 (54.3)	.87	9 (56)	

^a^Comparisons between the intervention and control groups for the trial were made using chi-square tests. *P*<.05 was considered significant.

^b^N/A: not applicable.

### How Students Gathered Information and Reached Diagnoses

From the qualitative think-aloud data, 4 major themes were identified relating to how students gathered information: data gathering strategies, structure of eCREST, diagnostic hypotheses, and confidence and uncertainty.

#### Theme 1: Data Gathering Strategies

Students had different data gathering goals and strategies for gathering information.

##### Being Thorough

Some students reported that they aimed to be thorough when gathering data and were aware of the potential pitfalls of the unpacking principle and not gathering all necessary information. Consequently, they asked many questions to reassure themselves that they had not missed any relevant information and symptoms indicative of serious disease. However, they acknowledged that this approach to data gathering could be lengthy and possibly led to them asking irrelevant questions:

I should probably go through those but I don’t know, I do feel like I like to be thorough and I do want to ask all of the questions.

P4

I think I could be more concise. Cos, I just kind of ask everything just in case.

P16

##### Being Focused

Other students reported that they aimed to be focused when investigating the patient’s symptoms and wanted to ensure that all the information they asked for was relevant to the patient case:

I don’t really want to ask any more of these I mean partly because I feel like...I want to be focused, so I’m not really going to ask the rest of these which are potentially not that related.

P10

##### Being Succinct

Some also described that they aimed to be succinct and limit the number of questions they asked the patient, possibly because the way eCREST was structured or perhaps to be more time efficient. This shows that some students were less aware of the potential negative consequences of the unpacking principle and the importance of gathering all relevant information:

Maybe I should have not tried to limit myself to a specific number and asked what I thought was actually appropriate.

P2

##### Random Selection

Some students reported that they were asking questions randomly and had no discernable reasoning behind the way they gathered information. Students may have adopted this seemingly random approach because they were less engaged with patient cases in eCREST:

So these questions I guess aren’t like very helpful but because they’re there I’m going to ask them anyway.

P11

#### Theme 2: Structure of eCREST

The way in which eCREST was structured appeared to be one mechanism by which eCREST influenced how students gathered information. Students perceived eCREST to have both positive and negative effects on how they gathered information.

##### Organized Data Gathering

Students reported that the structure of eCREST helped them organize the way they gathered information by asking students to chunk their history taking into sections and regularly visualizing their diagnostic hypotheses. This helped them to hone their questions toward their diagnoses and may have helped some students to overcome the unpacking principle to take a more focused approach:

I guess it’s good because it makes you streamline your thoughts regarding diagnosis after you have limited information available and I think that probably helps time management within GP settings, because it’s making you streamline your questions.

P9

I think it was useful even for me to just like, to see when I’m taking the history, I think that...I don’t really write down...the top five differential diagnosis when I’m taking history...Yeah, just better at visualizing it, and organizing it.

P13

Some appeared more focused in their approach to gathering data, as they thought of their own relevant questions first and then used eCREST as a checklist to confirm that they had not missed anything:

It’s probably useful to try and think about this before I look at the list.

P1

##### Unrealistic Data Gathering

However, others reported that the list of questions in eCREST led them to ask questions more randomly and less strategically than they would in a real consultation:

I think it just biased the way in which I asked the questions. Because I wouldn’t have just kind of gone through it and clicked on it as I went through.

P2

Some students felt that the lack of open questions in eCREST hindered their ability to gather information and felt open questions would have offered more relevant information in real life:

It’s quite useful to make you think about the questions but because I don’t ask questions in that, I feel like my own style is quite different to the way it’s set out here...I’d be quite like open with the patient. I’d be like “tell me more.” And then I’d be able, I’d have some better idea, I’d have a better timeline of the things.

P15

#### Theme 3: Diagnostic Hypotheses

The way in which eCREST guided how students generated and reassessed diagnostic hypotheses appeared to be another mechanism by which eCREST influenced how students gathered information.

##### Early Generation of Diagnostic Hypotheses

Some students found that eCREST helped them to think of diagnoses at an earlier stage than they usually would, which potentially helped them to avoid anchoring on a particular diagnosis early in the consultation and had an impact on the questions they asked:

Usually I don’t really think about differentials so early on in a consultation...so this encouraged me to rule out different diagnoses from a very early point.

P12

##### Cognitive Biases

Some students became fixated on one or two initial diagnoses (anchoring) and would consequently seek information to confirm the diagnosis and stopped investigating other causes of the symptoms (confirmation bias). This may explain why some students took a more succinct approach to gathering information:

[Question: what will you try to improve?] Consider everything the patient has said and I think just not try to make diagnosis fit, like the COPD that I was trying to make her fit.

P7

Two students showed awareness of confirmation bias during the task and made a conscious effort to seek other information but consequently may have asked too many irrelevant questions and led to students taking a thorough approach to gathering data:

I think I normally like to sort of focus on a system, so do almost a respiratory thing and then move on to cardiac...although I shouldn’t get too into confirming about, I’ll just ask about any other symptoms.

P4

Often I ask confirmatory questions...and just exclude things that I just know weren’t on my differential and so my differential didn’t really change.

P11

##### Reflection

A few students reported that they found it useful that eCREST gave them time to pause, think, and reflect on their diagnoses because in clinical practice this is not always possible. The opportunity to reflect may explain why students were able to demonstrate the use of focused data gathering strategies and avoid some cognitive biases in eCREST:

It’s nice to just click the questions, and then spend five minutes thinking about it. I think when you’re actually seeing patients there’s emphasis on it being slick.

P14

##### Alternative Diagnoses

There was evidence that eCREST helped students to avoid anchoring and confirmation bias, as most students reported that the prompts in eCREST to reassess diagnoses helped them to consider alternative diagnoses and reflect on the information they were gathering. This may have helped students to take a more focused approach to gathering data:

The fact that it makes you reconsider...your diagnosis after asking questions, asking a set number of questions is good practice for reality, when you should be doing that but you probably don’t.

P10

It makes you like re-evaluate your ranking of diagnosis because then you actually have think about the questions and why you’re asking them in the first place.

P10

However, some students demonstrated a random approach to reassessing their diagnoses and reported that the prompts to revise their diagnoses did not always help them to consider alternative diagnoses and stay open minded.

I’m just going to put in, arbitrarily, asthma...ischemic heart disease.

P8

A few students felt that the process of continually reviewing their diagnoses was too structured and some forgot information they gathered earlier in the case, which may have explained why their data gathering strategy appeared random:

It did perhaps make me think in that kind of modular way...each time I only considered the six questions that had been before. And forget about what had happened before that. So, like less of a kind of continuous set of questions and more like, oh, in the last six questions she said she like didn’t have a fever.

P16

#### Theme 4: Confidence and Uncertainty

The way in which eCREST influenced students’ confidence and level of uncertainty in their clinical decision-making skills appeared to be another potential mechanism by which eCREST influenced how students gathered information.

##### Creating Uncertainty

Some students reported that the structure of eCREST created some uncertainty, as the lists of diagnoses and questions encouraged them to reconsider diagnoses and think of the reasons behind choosing a diagnosis. This again suggests that the prompts to review diagnoses and reflect might have helped students to avoid anchoring and have a focused approach to gathering information from the patient:

I liked that there were all the differentials, you thought you knew but you still, it put that sort of seed of doubt in your mind. So in a sense...even though maybe I wasn’t as flawless in terms of safety netting and making sure I didn’t miss things, I think it forced you think okay well could it? And...constantly, by making you reconsider your diagnosis it did really make you think harder about each one.

P4

##### Managing Uncertainty

Some students talked about needing more confidence in their decision making to help them manage their uncertainty when gathering information from the patient:

Maybe I should just have more confidence in saying that, okay this is what I think and it’s still consistent you know.

P12

Students also recognized a tension between making confident judgments about the most likely diagnosis and fearing serious consequences for the patient if they missed a more serious diagnosis. This may explain why some students adopted a thorough approach to gathering information and potentially used this approach to manage their uncertainty. There was also evidence from students’ reflections that they were starting to manage this uncertainty by taking a more focused approach to gathering data and using safety netting:

You can give every investigation and then be sure, but actually realistically when you’re trying to think well I can’t get every single blood test in the world, this is the initial management...at the moment I feel I have to be quite brave, because you think well what if I do miss something that’s terrible? But then I suppose it’s easy to think okay, well what are the absolute terrible things? Make sure that I don’t miss those, so for example an x-ray would cover a lot of bases in a sense.

P4

Learning to be comfortable...with a degree of uncertainty is important to GP and several other specialties. I will need to learn from more experience how much safety netting and investigation for other possible differentials is appropriate.

P3

Some students felt that eCREST and simulations were a way to practice taking responsibility for decisions and managing their uncertainty. It was suggested that being more engaged with the simulation helped students to adopt more focused or thorough data gathering patterns and avoid the unpacking principle, as it made them feel more responsible for the *patient* and motivated them to not miss any serious conditions:

I think every time I do...cases like this, and certainly when I see real patients, like I’m on GP at the moment...I’m going to try and think about it, to approach it as though I was the GP seeing the patient alone, and you’re their only point of care...and therefore fully responsible for them. Which forces you to really think carefully about differentials and things not to miss.

P4

Some students felt reluctant to make decisions they would not be responsible for in real life and were perhaps less engaged with the simulation and the opportunity to practice managing uncertainty:

I would probably review him in the week, oh wait, actually, I don’t know. I want to consult with my senior first but it’s hard to say. I mean just to be...I mean if, in real life given, if I don’t have any senior to talk to and if I don’t know whether he has any back pain just to be safe I would probably just refer him, just to be sure, I guess.

P13

A few students felt that investigating rare and unlikely diagnoses was unrealistic and were perhaps less engaged with the simulation. This may have led to some students adopting more succinct data gathering approaches that just focused on common illnesses and led them to be more susceptible to cognitive biases:

I appreciate that...what they’ve said about all of their diagnoses but...in real life, what happens is: you make a working diagnosis, and everything else is left behind—you don’t continue those, generally. There might be some things you safety net, but by and large, when it’s clear cut—as that was—you would almost take that, go with it, do a few things, just to be sure. Where I am—and I know this is a GP situation, but in A&E, if you said, ‘Oh, my fifth diagnosis of this patient is “Addison’s Disease,”’ I think you’d...be laughed out of the department, realistically.

P1

### Identifying Data Gathering Patterns

**Figure 2:**
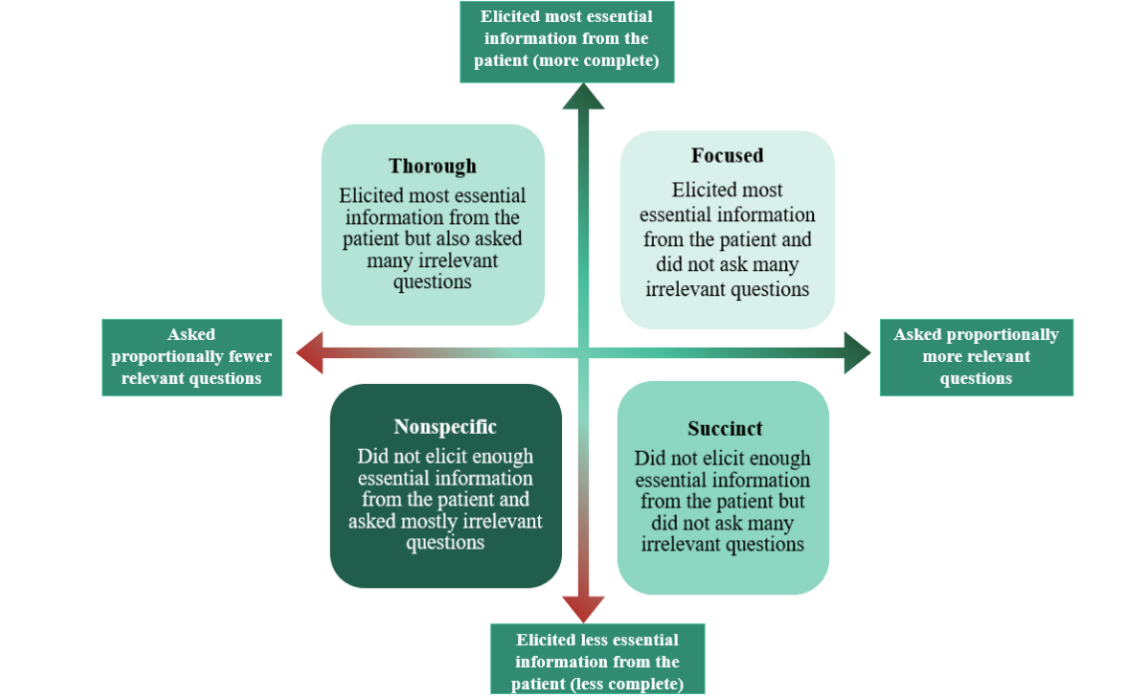
Characteristics of data gathering patterns based on the “Essential” and “Relevant” variables.

The qualitative data showed that students aimed to apply 3 distinct data gathering strategies while using eCREST. It suggested that these strategies may manifest in the quantitative data as different data gathering behaviors and indicated how eCREST might influence them. As described in the *Methods* section, we sought to observe these data gathering patterns in the trial sample by using trial students’ scores on the Essential and Relevant variables. Figure 2 summarizes the characteristics of the data gathering patterns. Those who displayed Focused and Thorough patterns tended to elicit the most essential information and take a more complete patient history. However, those who showed Thorough patterns also tended to gather more irrelevant information. Those who displayed a Succinct pattern or a Nonspecific pattern did not elicit enough essential information from the patient and took a less complete history from the patient. However, those who showed Succinct patterns also did not gather much irrelevant information.

**Figure 3:**
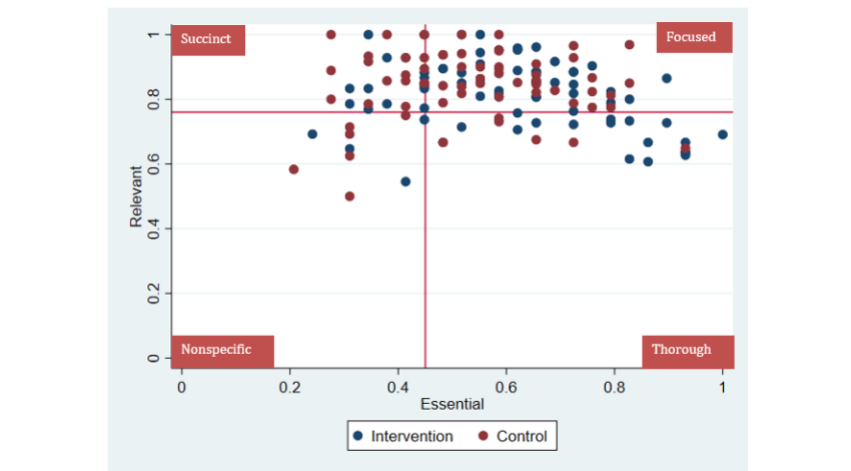
Scatter plot showing students’ scores on the “Essential” and “Relevant” clinical reasoning measure in the trial.

Figure 3 shows a scatter plot of the trial students’ scores on the Essential and Relevant variables by the trial group and whether their scores fell on or below the lowest quartile for each variable, which determined the data gathering pattern they were identified as displaying. The data show most students displayed Focused patterns, but a significant proportion displayed other patterns and the prevalence appeared to differ by the trial group.

### Impact of eCREST on Data Gathering Patterns

In the trial, there was a significant difference between the intervention and control groups in the type of data gathering pattern used (*χ*^2^_3_=9.9; *P*=.02; Table 2). Those in the intervention group were much more likely to show a Thorough pattern compared with the control group (21/78, 27% vs 6/70, 9%) but less likely to demonstrate a Succinct pattern (13/78, 17% vs 20/70, 29%). The likelihood of showing Focused or Nonspecific patterns were similar between the intervention and control groups (40/78, 51% vs 38/70, 54% and 4/78, 5% vs 6/70, 9%, respectively).

**Table 2:** Data gathering patterns observed in the electronic Clinical Reasoning Educational Simulation Tool in the trial data.

Data gathering pattern^a^	Intervention (n=78), n (%)	Control (n=70), n (%)	Total (N=148), n (%)
Thorough	21 (27)	6 (9)	27 (18.2)
Succinct	13 (17)	20 (29)	33 (22.3)
Focused	40 (51)	38 (54)	78 (52.7)
Nonspecific	4 (5)	6 (9)	10 (6.8)

^a^Patterns were significantly associated with the trial group; *χ*^2^_3_=9.9, *P*=.02 (as in the main text of the paper).

## Discussion

### Principal Findings

This study identified a range of data gathering patterns that students applied when generating data from virtual patients. The qualitative data indicated how eCREST can help students to take a more thorough approach, and potentially reduce the impact of cognitive biases, through continuous revision of diagnoses and allowing students to practice managing their uncertainty. Quantitative data from the trial indicated that eCREST influenced students to demonstrate more Thorough data gathering patterns.

This study showed how virtual patients such as eCREST can be used to address the cognitive biases of the unpacking principle, confirmation bias, and anchoring by continuously prompting students to reflect throughout a patient consultation. The qualitative data showed that these prompts encouraged many students to investigate patients more thoroughly and re-evaluate their diagnoses. This may have helped them to overcome the potentially negative consequences of these biases, such as missing serious diagnoses [32,47]. The trial data also indicated that students who had used eCREST before exhibited more Thorough data gathering patterns than controls, which may have helped them to address these biases [33]. We have no empirical data to suggest that any data gathering pattern is better than another and in which clinical circumstances they might be most appropriate. In clinical practice, a more Focused approach where most of the important information is gathered without gathering too much irrelevant information may be ideal, particularly given time constraints in real consultations. Policy and clinical guidelines in the United Kingdom and elsewhere are increasingly recommending more focused and thorough investigations of patients to avoid missing red flag symptoms, particularly in primary care for serious conditions such as cancer [8,25]. Thus, the data gathering pattern that eCREST is encouraging is in line with recommendations from health policy and may be particularly appropriate for the investigation of conditions such as cancer.

A unique contribution of this study is the use of a mixed methods approach. The qualitative data showed distinct data gathering strategies that students aimed to undertake and how eCREST influenced how they gathered information. We gained insight into students’ rationales for their data gathering strategies. Those who reported wanting to be Thorough explained that they used this strategy to avoid missing key information about a serious diagnosis and because they felt uncertain about the case. The students who reported wanting to be more focused verbalized the importance of asking only relevant questions. Those who aimed to be more succinct reported wanting to limit the information they gathered. This was perhaps because in real consultations, students would only have limited time with a patient, but this may have led them to be susceptible to biases and miss symptoms indicative of a serious disease. We also found that some students were less engaged with eCREST and randomly clicked on questions and made decisions. Further research is needed to understand which students might not benefit from clinical reasoning teaching delivered via virtual patients and how it can be further adapted to students’ needs.

Similar to previous literature on clinical reasoning, we identified a central theme for managing uncertainty [48,49]. The qualitative data showed how eCREST created uncertainty by prompting students to reconsider diagnoses. It also offered students an opportunity to practice managing their uncertainty by conducting thorough investigations and safety netting for the worst-case scenarios [48]. Therefore, eCREST may have helped to calibrate students’ confidence. Previous studies have shown that there is little correlation between confidence and diagnostic accuracy in students or physicians and that overconfidence increases with more difficult cases [49-51]. Given that the cases in eCREST were relatively complex for students, it is perhaps a positive result that many students reported uncertainty and a lack of confidence while making diagnostic decisions in eCREST, suggesting that it might help reduce overconfidence in difficult cases.

This study also showed the potential impact of virtual patients, such as eCREST, on medical education. Given the increasingly limited exposure to real patients, virtual patients can provide students with some form of clinical experience [9]. They also provide an opportunity for educators to observe in real time students’ data gathering strategies and patterns of behavior and inform the formative assessment of students’ clinical reasoning skills. Previous research in education has used inventories to identify students’ approaches to studying, such as the Approaches and Study Skills Inventory for Students [52-55]. Approaches and Study Skills Inventory for Students helps educators and students to identify general patterns on how students approach learning in certain circumstances and allows educators or computer programs to offer advice on other approaches that could be used. Similarly, educators could use data from eCREST in a reflective way and as a basis to provide feedback on data gathering patterns that might help students to improve their reasoning [21].

### Limitations

Our study was limited to medical students, but we are undertaking further research with a range of health care professionals and students to understand how eCREST can be used more widely in clinical education. Students volunteered to take part in the trial and think-aloud study; therefore, this sample might have been different from those who did not volunteer, leading to possible selection bias. In addition, in the think-aloud study, students varied in their ability to verbalize their thoughts and the sample was not representative; therefore, it is likely that not all patterns and rationales for investigations were identified [56,57]. In common with all think-aloud and interview study designs, participants were observed and prompted to speak; therefore, students may have attuned their responses because of social desirability, and they were more reflective than they would have been if unprompted or unobserved [56,57].

### Conclusions

This study found that students displayed a variety of data gathering patterns while using virtual patients. Data from the trial indicated that virtual patients such as eCREST might influence students to be more thorough in their data gathering. The think-aloud interviews suggested the mechanisms by which eCREST influenced students included helping them to continuously reflect on their diagnoses and manage uncertainty. These findings suggest that virtual patients, which increase the thoroughness of data gathering, could help future physicians to reduce missed diagnostic opportunities during future consultations. Virtual patients could also provide medical educators with a more accessible way of observing and identifying students’ data gathering patterns, which may enable them to provide more tailored feedback on reasoning. Further research is needed to understand how data gathering patterns relate to existing clinical and pedagogical practices and vary across clinical contexts.

## Appendix

Multimedia Appendix 1 Flowchart of the procedure for the trial.

## Abbreviations

eCREST: electronic Clinical Reasoning Educational Simulation Tool
