# Supplementary figures and images for: Using Virtual Patients to Explore the Clinical Reasoning Skills of Medical Students: Mixed Methods Study
Source: J Med Internet Res. 2021 Jun 4;23(6):e24723. doi: 10.2196/24723 (PMC8214179; doi:10.2196/24723)

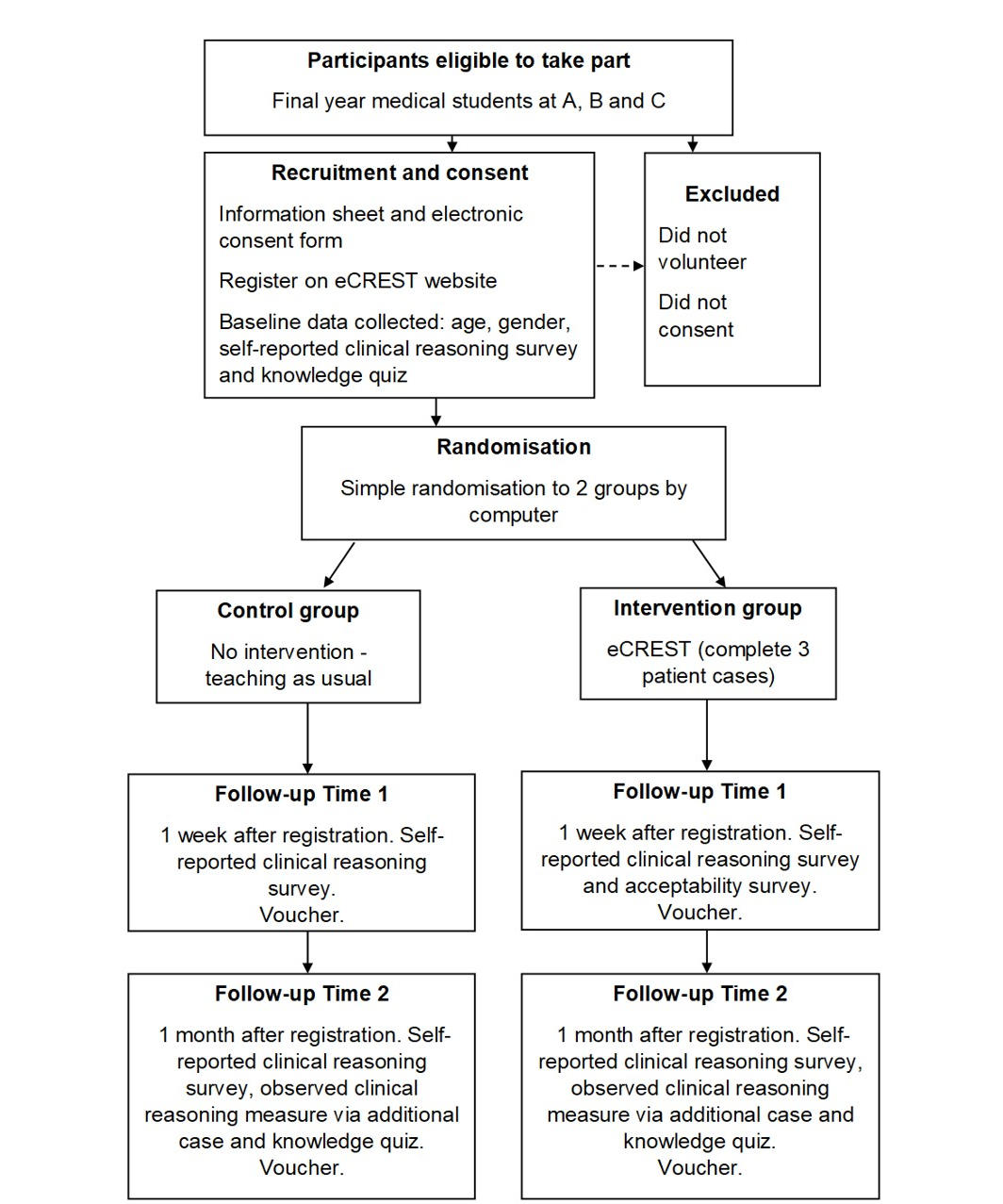

Supplement: Multimedia Appendix 1 [file jmir_v23i6e24723_app1.png]
